# TLR9 acts as a sensor for tumor-released DNA to modulate anti-tumor immunity after chemotherapy

**DOI:** 10.1186/s40425-019-0738-2

**Published:** 2019-10-16

**Authors:** Tae Heung Kang, Chih-Ping Mao, Young Seob Kim, Tae Woo Kim, Andrew Yang, Brandon Lam, Ssu-Hsueh Tseng, Emily Farmer, Yeong-Min Park, Chien-Fu Hung

**Affiliations:** 1 Department of Immunology, College of Medicine, Konkuk University, 268, Chungju, South Korea; 2 MD-PhD Program, Johns Hopkins University, School of Medicine, Baltimore, MD USA; 3 Graduate Program in Immunology, Johns Hopkins University, School of Medicine, Baltimore, MD USA; 4 Department of Pathology, Johns Hopkins University, School of Medicine, Baltimore, MD USA; 5 Division of Infection and Immunology, Graduate School of Medicine, Korea University, Seoul, South Korea; 6 MD-PhD Program, Baylor College of Medicine, Houston, TX USA; 7 Department of Oncology, Johns Hopkins University, Baltimore, MD USA

**Keywords:** Toll-like receptor 9, Tumor DNA, Chemotherapy

## Abstract

The tumor microenvironment exists in a state of dynamic equilibrium, in which a balance of agonist and antagonist signals govern the anti-tumor immune responses. Previous studies have shown that chemotherapy could shift this balance in favor of agonistic signals for the anti-tumor immune responses mounted by CD8+ cytotoxic T lymphocytes (CTL), providing sufficiently high antigen density within the tumor. We undertook the current study to characterize the anti-tumor immune response following chemotherapy and its underlying mechanisms. We show that this ‘adjuvant effect’ of chemotherapy is, at least partially, mediated by the release of tumor DNA and acts through the Toll-like receptor 9 (TLR9) pathway. We found that tumor-released DNA causes accumulation, antigen uptake, and maturation of dendritic cells (DCs) in the tumor in a TLR9-dependent manner. These DCs subsequently migrate into the draining lymph nodes and prime tumor-specific CTLs. Our study provides novel insights to the molecular and cellular mechanisms by which chemotherapy converts the tumor microenvironment into a site permissive for the activation of a potent tumor-specific adaptive immune response.

## Introduction

The adaptive immune system contributes to the control of cancer [1]. In particular, the ability of CD8^+^ cytotoxic T lymphocytes (CTLs) to mount a rapid, robust, and specific response against tumor cells at multiple sites in the body has promoted the idea that the immune system can be harnessed through vaccination to eradicate metastasis or to prevent disease relapse, which are the predominate causes of mortality due to cancer [2–4]. Nonetheless, strategies to enhance the CTL-mediated anti-tumor immune response via direct vaccination of tumor antigens have had limited clinical success thus far [5].

A potential explanation for these findings is the complexity and diversity of the tumor microenvironment (TME). Particularly, many tumors have been immunologically described as “cold tumor”, characterized by the lack of antigen presentation, immune response generation, and/or tumor CTL infiltration [6]. Significant research efforts have thus focused on developing therapeutic strategies capable of converting these “cold tumor” into “hot tumor” that are more susceptible to subsequent clearance by anti-tumor immunity [7]. Previous studies have reported the generation of tumor-specific immune responses in tumor-bearing mice cured via chemotherapy treatment, and that the chemotherapy-cured mice are capable of rejecting subsequent challenges with the same tumor [8–10]. Similarly, we have explored the effect of chemotherapy on the adaptive immune response in the TME, and found that a wide spectrum of pharmacologic agents applied in chemotherapy could convert this microenvironment into a site favoring the activation of tumor-specific CTLs, provided that there is a sufficiently high antigen density within the tumor [11].

We undertook the current study to further characterize the anti-tumor immune response following chemotherapy and its underlying mechanisms. We show here that host Toll-like receptor 9 (TLR) acts as a sensor for extracellular DNA shed from dying tumor cells and is critical for the adjuvant effect of chemotherapy. We found that TLR9 signaling triggers the accumulation, maturation, and lymph node migration of antigen-loaded tumor dendritic cells (DCs). Within the lymph nodes, these DCs mediate activation of tumor-specific CTLs, which proliferate and traffic into the tumor to control cancer growth.

## Results

### Tumor DNA is released into circulation after chemotherapy and facilitates the generation of anti-tumor immune response

**Fig. 1:**
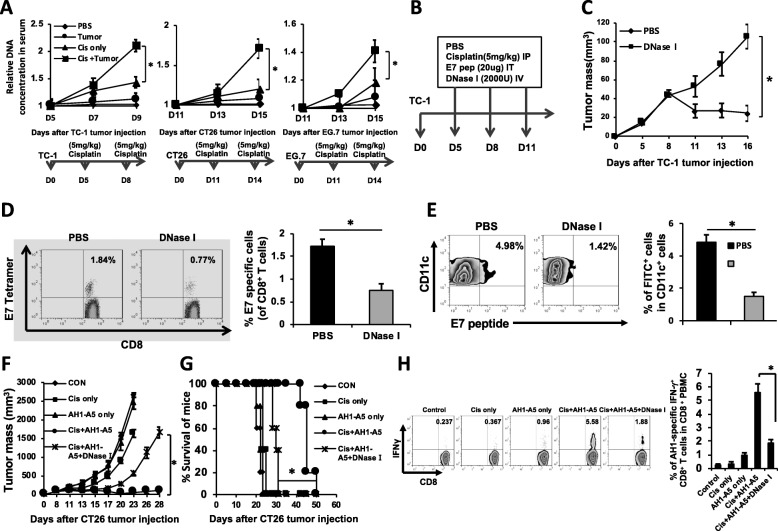
Effect of chemotherapy induced tumor DNA release on the anti-tumor immune response following chemotherapy. **a**) Quantification of DNA released from tumor cells in vivo with or without cisplatin (*n* = 5). **b-e** TC-1 tumor-bearing C57BL/6 mice were treated with cisplatin intraperitoneally, together with intratumoral injection of unlabeled **(c-d)** or FITC-labeled **(e)** E7 peptide. Mice were then administered with either DNase I or PBS. **b** Schematic diagram. **c** Line-graph depicting tumor growth kinetics in DNase I-treated compared to PBS-treated mice (*n* = 5). **d** PBMCs were collected from mice, stained with E7-D^b^ tetramer, and examined by flow cytometry. Left: Representative flow cytometry depicting the frequency of E7-specific CTLs. Right: Bar graph quantification (*n* = 5). **e** Draining lymph nodes were processed into single cells and stained for CD11c. Left: Representative flow cytometry depicting the frequency of E7-loaded tumor DCs in the draining lymph nodes. Right: Bar graph quantification (*n* = 5). **f-h** CT26 tumor-bearing BALB/c mice were treated with cisplatin intraperitoneally, together with direct AH1-A5 peptide injection into the tumor. Mice were co-treated with either DNase I or PBS. **f** Line-graph depicting tumor growth kinetics (*n* = 5). **g** Kaplan-Meier survival analysis of mice (*n* = 5). **h** PBMCs were collected, pulsed ex vivo with AH1-A5 peptide, and co-stained the next day for CD8 and IFN-γ. Left: Representative flow cytometry depicting the number of systemic AH1-A5-specific CTLs. Right: Bar graph quantification (*n* = 5). Significance determined by student’s *t* test (**a**, **c-e**, & **h**) or ANOVA (**f-g**). Data are represented as mean ± SD. *P < 0.01

Accumulating evidence indicates that stressed or dying tumor cells that are exposed to chemotherapy can release various cellular contents that contribute to the subsequent generation of anti-tumor immune response, including immunostimulatory chaperone proteins [12] and neoantigenic peptides [13]. While tumor released DNA has been widely utilized as an important material for tumor detection and monitoring [14], few studies have explored the involvement of tumor released DNA in the generation of anti-tumor immunity following chemotherapy. We sought to evaluate the potential contribution of tumor-derived circulating DNA on the elicitation of anti-tumor immunity. To this end, we first examined the release of tumor-DNA following tumor cell death induced by chemotherapy. We observed gradual accumulation of DNA in the serum of mice bearing TC-1, CT26, or EG7 tumors following cisplatin treatment (Fig. 1a), suggesting that chemotherapy causes systemic release of tumor DNA into circulation. To test whether tumor released DNA plays a role in the generation of anti-tumor immune response following chemotherapy, we treated TC-1 tumor-bearing mice with intraperitoneal cisplatin and intratumoral HPV16-E7 (E7) peptide injections, followed with intravenous administration of either DNase I or PBS (Fig. 1b). TC-1 tumor-bearing, cisplatin and E7 peptide treated mice that also receive DNase I injection failed to control the growth of tumor as compared to those that received PBS injection (Fig. 1c). Intriguingly, DNase I injection also led to a reduction in the abundance of systemic E7-specific CTLs and E7-presenting CD11c + DCs in regional lymph nodes (Fig. 1d-e). To confirm these data in a different model, we treated CT26 tumor-bearing BALB/c mice with cisplatin intraperitoneally together with intratumoral AH1-A5 peptide injection, with or without systemic DNase I injection. DNase I administration led to poor control of tumor progression (Fig. 1f-g) and markedly weakened the immune response generated by cisplatin and AH1-A5 peptide treatment (Fig. 1h). These data show that chemotherapy causes systemic release of tumor DNA into circulation, which has an important role in facilitating the subsequent generation of effective anti-tumor immune response.

### Host TLR9 is critical for the generation of anti-tumor immune response after chemotherapy

**Fig. 2:**
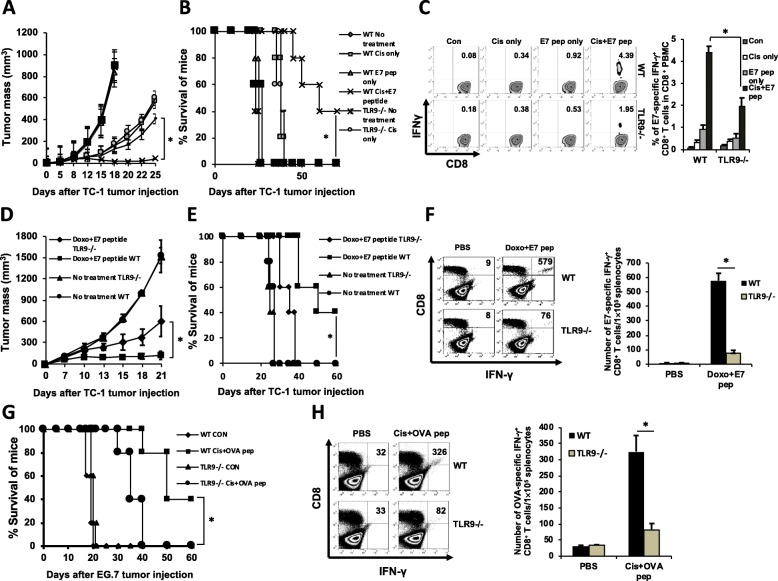
Effect of TLR9 on the anti-tumor immune response following chemotherapy. **a-c** TC-1 tumor-bearing wildtype or TLR9^−/−^ mice were treated with indicated combinations of cisplatin and/or E7 peptide. **a** Line-graph depicting tumor growth kinetics (*n* = 10). **b** Kaplan-Meier survival analysis of mice (*n* = 10). **c** Left: Representative flow cytometry depicting the frequency of systemic E7-specific CTLs in TC-1 tumor-bearing mice (*n* = 5). Right: Bar graph quantification. **d-f** TC-1 tumor-bearing wildtype C57BL/6 or TLR9^−/−^ mice were treated with doxorubicin and E7 peptide. **d** Line-graph depicting tumor growth kinetics (*n* = 10). **e** Kaplan-Meier survival analysis of mice (*n* = 10). **f** Left: Representative flow cytometry depicting the frequency of systemic E7-specific CTLs (*n* = 5). Right: Bar graph quantification. **g-h** EG7 lymphoma-bearing wildtype or TLR9^−/−^ mice were treated with cisplatin and Ova peptide or with PBS control. **g** Kaplan-Meier survival analysis of mice (*n* = 5). **h** Left: Representative flow cytometry depicting the frequency of systemic Ova-specific CTLs in mice (*n* = 5). Right: Bar graph quantification. Significance determined by ANOVA (**a-b**, **d-e**, **g**), student’s *t* test (**c**, **f**, **h**). Data are represented as mean ± SD. *P < 0.01

We sought to determine the process by which chemotherapy-induced tumor DNA release contribute to the generation of antigen-specific anti-tumor immune response. In this regard, several DNA sensor proteins have been identified and linked to the immunogenic recognition of DNA [15]. Among the various DNA sensor proteins, synthetic agonists targeting the TLR9 signaling pathway have been widely explored as methods to enhance the immunogenicity of anti-cancer therapy [16], however, it remains unclear whether tumor released DNA can act as an endogenous TLR9 agonist to trigger the generation of an anti-tumor immune response following chemotherapy. We thus decided to explore the role of TLR9 in this process by inoculating either wildtype C57BL/6 or TLR9^−/−^ mice with TC-1 tumor cells. After the tumor was established, we treated mice with cisplatin intraperitoneally and with E7aa43–62 peptide by intratumoral injection. Exogenous E7 peptide was administered into the tumor because the endogenous expression of E7 in TC-1 cells is low, and we previously reported that high antigen density within the tumor is critical for the generation of an anti-tumor immune response after chemotherapy [11]. Combined chemotherapy and vaccination led to persistent control of tumor growth in wildtype mice but not in their TLR9^−/−^ counterparts (Fig. 2a-b). Because TC-1 cells carry the wildtype TLR9 allele, loss of tumor control in TLR9^−/−^ mice must be due to a host-intrinsic requirement for TLR9. E7-specific CTL response were markedly reduced in TLR9^−/−^ TC-1 tumor-bearing mice treated with chemotherapy and E7 peptide injection compared to wildtype mice (Fig. 2c), suggesting that host TLR9 influences the adaptive immune response generated by chemotherapy. We also observed consistent results in TC-1 tumor-bearing mice administered with doxorubicin (Fig. 2d-f) and in mice inoculated with a different tumor type, EG7, a lymphoma model carrying the ovalbumin (Ova) antigen (Fig. 2g-h). Altogether, these data show that host TLR9 is essential for the anti-tumor immune response following chemotherapy.

### TLR9 mediates accumulation, antigen uptake, lymph node migration, and maturation of tumor DCs after chemotherapy

**Fig. 3:**
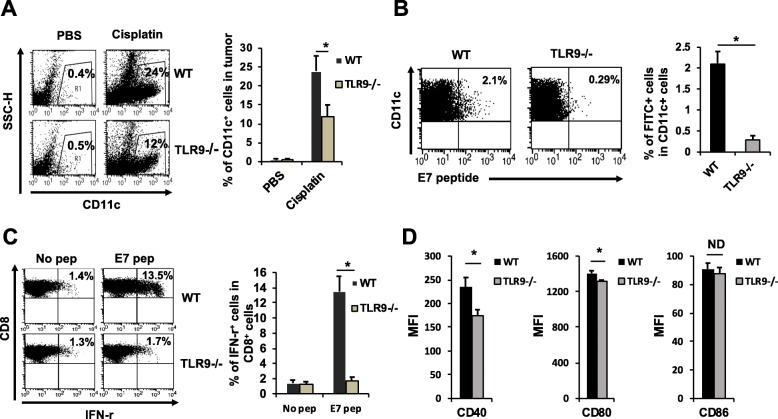
Role of TLR9 in the accumulation, trafficking, antigen presentation and maturation of tumor DCs after chemotherapy. **a** TC-1 tumor-bearing wildtype or TLR9^−/−^ mice were treated intraperitoneally with cisplatin or PBS. Tumor-infiltrating cells were stained for CD11c and examined by flow cytometry to detect the number of tumor DCs. Left: Representative flow cytometry depicting the frequency of tumor DCs. Right: Bar graph quantification (*n* = 5). **b-c** TC-1 tumor-bearing wildtype of TLR9^−/−^ mice were treated with cisplatin intraperitoneally, together with injection of FITC-labeled (**b**) or unlabeled (**c**) E7 peptide into the tumor. **b** Cells from draining lymph nodes were stained for CD11c and examined by flow cytometry. Left: Representative flow cytometry depicting the frequency of E7-loaded tumor CD11c^+^ DCs. Right: Bar graph quantification (*n* = 5). **c** DCs were purified from lymph nodes and co-incubated with E7-specific CTLs. Cells were stained for IFN-γ and examined by flow cytometry (*n* = 5). Left: Representative flow cytometry depicting activation of E7-specific CTLs. Right: Bar graph quantification. **d** TC-1 tumor-bearing mice were treated intraperitoneally with cisplatin. Tumor-infiltrating cells were harvested and co-stained for CD11c and for CD40, CD80, or CD86, and examined by flow cytometry. Bar graph indicates expression status (as mean fluorescence intensity (MFI)) of CD40, CD80, or CD86 on CD11c^+^ tumor DCs (*n* = 5). Significance determined by student’s *t* test. Data are represented as mean ± SD. *P < 0.01, ND = no difference

We next looked into the mechanisms by which TLR9 contributes to the anti-tumor immune response after chemotherapy. Since TLR9 is predominately found on professional antigen presenting cells (APC) [17], we examined the influence of TLR9 on tumor DCs. After chemotherapy, the frequency of DCs within the tumor of wildtype mice increased by 60-fold (Fig. 3a). We next examined the ability of these DCs to uptake antigen and travel into regional lymph nodes. We treated wildtype or TLR9^−/−^ TC-1 tumor-bearing mice with cisplatin intraperitoneally together with FITC-labeled E7 peptide by intratumoral injection. After 2 days, there were 10 times more FITC^+^ DCs in the tumor draining lymph nodes of wildtype mice relative to their TLR9^−/−^ counterparts, suggesting that TLR9 signaling is critical for migration of antigen-loaded tumor DCs into regional lymph nodes (Fig. 3b). Moreover, DCs purified from tumor draining lymph nodes of wildtype mice were able to stimulate E7-specific CTLs 10 times more efficiently than DCs from TLR9^−/−^ mice (Fig. 3c). Furthermore, we examined the expression of co-stimulatory molecules on tumor DCs from TC-1-bearing wildtype or TLR9^−/−^ mice treated with cisplatin. Tumor DCs from wildtype mice had higher average expression of CD40 and CD80 compared to DCs from TLR9^−/−^ mice (Fig. 3d), suggesting that host TLR9 promotes the maturation of tumor DCs. These data indicate that TLR9 signaling leads to an accumulation of DCs within the TME and triggers their maturation and migration into the regional lymph nodes, where they can prime tumor-specific CTLs.

## Discussion

In this study, we found that host TLR9 acts as a sensor for tumor DNA that modulates the anti-tumor immune response following chemotherapy. Particularly, we showed that TLR9 promotes the maturation and migration of antigen-presenting DCs from the TME to the regional lymph nodes, where they subsequently activate tumor-specific CTLs leading to effective tumor control. As mentioned previously, over 10 DNA sensor proteins, in addition to TLR9, have been identified and linked to the immunogenic recognition of DNA [15]. Many of these DNA sensors have been shown to contribute to the initiation of innate immune responses following chemo- or radiation therapy by sensing cytosolic DNA accumulated in stressed tumor cells leading to tumor secretion of pro-inflammatory cytokines via the STING signaling pathway [18, 19]. Our current finding, coupled with existing literatures, suggests that multiple tumor DNA sensing pathways may be simultaneously involved in the stimulation of anti-tumor immune response following chemotherapy in both animal models as well as in cancer patients. Also, our finding that TLR9 deficient mice fail to induce effective antitumor immune response following chemotherapy provides a potential explanation for the variations of the immune adjuvanting effects of cancer chemotherapy observed in the clinical settings.

In addition to TLR9-mediated tumor DNA sensing, we have previously found that TLR4 also promotes activation of tumor-specific CTLs after chemotherapy by recognizing the chromatin-associated factor HMGB1 released from dying tumor cells [11]. In addition to DNA and protein content, it is possible that chemotherapy may also trigger the release of RNA from dying tumor cells, which can in turn serve as a ligand for TLR7 or TLR8 [20–22]. Alternatively, Sistigu et al. have demonstrated that release of tumor-RNA induced by anthracyclines stimulates an antitumor immune response through TLR3 signaling [23]. Furthermore, Ganguly et al. have reported that RNA sequences can be complex with the antimicrobial peptide LL37 to trigger the activation and IFN-α, TNF-α, and IL-6 secretion by DCs [24]. Thus, it will be of interest to determine if, like tumor-released DNA, tumor-released RNA can also facilitate the adjuvant effect of chemotherapy by behaving as an agonist of TLR7 or TLR8 signaling. We infer that multiple types of ligands released by tumor cells following chemotherapy (e.g., tumor DNA, HMGB1, tumor RNA) may act through their respective TLRs to drive DC maturation and activation of tumor-specific CTLs.

## Materials and methods

### Mice

6- to 8-week old female C57BL/6 and BALB/c mice were purchased from the National Cancer Institute (Frederick, MD). TLR9^−/−^ mice [25] were purchased from the Mutant Mouse Regional Resource Center (Bar Harbor, ME). All animal procedures were performed in accordance with protocols approved by the Johns Hopkins Institutional Animal Care and Use Committee and in accordance with recommendations for the proper use and care of laboratory mice.

### Cells

Generation of TC-1 tumor cell line [26] and HPV16-E7-specific CTLs (recognizing epitope aa49–57 of E7) [27] has been described previously. EG7 cells (a derivative of EL4 lymphoma cells transduced with Ova) and CT26 (mouse colon carcinoma line) were obtained from ATCC (Manassas, VA). Cells were authenticated by short tandem repeat DNA fingerprinting. Cells were maintained at 37 °C under 5% CO2 atmosphere in RPMI-1640 medium supplemented with 10% fetal bovine serum, 50 U/ml penicillin/streptomycin, 2 mM L-glutamine, 1 mM sodium pyruvate, and 2 mM non-essential amino acids.

### Quantification of DNA concentration

For in vivo DNA concentration measurement in the TC-1 model, 10^5^ TC-1 cells were inoculated subcutaneously into C57BL/6 mice (5 per group). At days 5 and 8 after tumor challenge, naïve or TC-1 tumor-bearing mice were treated intraperitoneally with cisplatin (5 mg/kg) or PBS control. At days 5, 7, and 9 after tumor challenge, serum was collected from mice, and DNA concentration was determined with the Quant-iT PicoGreen dsDNA kit (Invitrogen, Carlsbad, CA).

For in vivo DNA concentration measurement in the CT26 or EG7 model, 10^6^ CT26 or EG7 cells were inoculated subcutaneously into BALB/c or C57BL/6 mice (5 per group), respectively. After 11 or 14 days, naïve or tumor-bearing mice were treated intraperitoneally with cisplatin (5 mg/kg) or PBS control. At days 11, 13, and 15 after tumor challenge, serum was collected from mice, and DNA concentration was determined with the Quant-iT PicoGreen dsDNA kit.

### Tumor treatment experiments

For experiments in the TC-1 model, TC-1 cells (1 × 10^5^ per animal) were inoculated subcutaneously into C57BL/6 or TLR9^−/−^ mice (10 per group). On days 5, 8, and 11 after tumor challenge, mice were administered with 5 mg/kg of cisplatin or doxorubicin intraperitoneally, with or without concurrent intratumoral injection of 20 μg of E7 peptide (aa43–62). PBS administrations were used as controls. Tumor growth was monitored by palpation and visual inspection twice per week. For experiments involving the use of DNase I, 2000 U of DNase I (Invitrogen, Carlsbad, CA) or PBS control were injected intravenously in concurrent with cisplatin and E7 peptide administration on days 5, 8 and 11 after tumor challenge.

For experiments in the CT26 model, CT26 tumor cells (2 × 10^5^ per animal) were inoculated subcutaneously into BALB/c mice (10 per group). On days 5, 8, and 11 after tumor challenge, mice were treated intratumorally with 20 μg of AH1-A5 peptide (SPSYAYHQF), intraperitoneally with cisplatin (5 mg/kg body weight), and/or 2000 U of DNase I intravenously. PBS injections were used as controls. Tumor growth was monitored by palpation and visual inspection twice per week.

For experiments in the EG7 model, EG7 tumor cells (2 × 10^6^ per animal) were inoculated subcutaneously into C57BL/6 or TLR9^−/−^ mice (10 per group). At 10, 13, and 16 days post tumor challenge, mice were administered with cisplatin (5 mg/kg) or PBS intraperitoneally, together with direct Ova peptide (20 μg) (aa241–270, SMLVLLPDEVSGLEQLESIINFEKLTEWTS) injection into the tumor. Tumor growth was monitored by palpation and visual inspection twice per week.

### Quantification of antigen-specific T cells

PBMCs were collected 1 week after the last drug/peptide injection. Erythrocytes were lysed in ammonium chloride-potassium bicarbonate buffer, and leukocytes were pulsed ex vivo with relevant peptide (1 μg/ml) (e.g., E7 aa49–57, Ova aa258–265, or AH1 aa6–14) overnight in the presence of Brefeldin A (BD Biosciences). Cells were stained with PE-labeled α-CD8 mAb (BD Biosciences), fixed and permeabilized with Cytofix/Cytoperm reagent (BD Biosciences), and then stained with FITC-labeled anti-IFN-γ mAb (BD Biosciences). The frequency of IFN-γ^+^ CLTs was examined by flow cytometry via FACSCalibur device (BD Biosciences), as previously described [28]. For tetramer binding analysis, PBMCs were co-stained with FITC-labeled anti-CD8 mAb (BD Biosciences) and PE-labeled H-2D^b^ tetramer loaded with HPV-16 E7 epitope (aa49–57; RAHYNIVTF) (Beckman Coulter, Hialeah, FL), and then examined by flow cytometry. For analysis of tumor-infiltrating E7-specific CTLs, tumor tissue was excised from tumor-bearing mice, minced, and passed through a 100 μm strainer. Single cells were co-stained with FITC-labeled α-CD8 mAb and PE-labeled E7-D^b^ tetramer and examined by flow cytometry. All data analysis was performed on gated lymphocyte populations (as defined by FSC/SSC features) using FlowJo software (Tree Star, Ashland, OR).

### Analysis of APCs

To monitor the effects of cisplatin on infiltration of APCs into the tumor, 10^5^ TC-1 cells were inoculated subcutaneously into wildtype or TLR9^−/−^ C57BL/6 mice (5 per group). On days 5 and 8 following tumor challenge, mice were administered intraperitoneally with cisplatin (5 mg/kg) or PBS control. 24 h after the final drug injection, tumor tissue was excised. To process excised tumor tissue into single cells, excised tumor tissues were minced and washed 2 times with PBS and then digested with dispase (500 U/ml) (Godo Shusei, Tokyo, Japan) at 37 °C for 20 min. Fragments were centrifuged at 150×*g* for 5 min; the supernatant was then discarded, and the pellet was resuspended in 5 ml of PBS and homogenized to single cells. The cells were then passed through a 100 μM mesh stainless wire sieve and washed 2 times with 20 ml of PBS. Cells were then resuspended in PBS and stained with APC-labeled anti-CD11c mAb (BD Pharmingen, San Diego, CA). To detect maturation of APCs, cells were co-stained with FITC-labeled anti-CD40, CD80, or CD86 mAb (BD Pharmingen) and then examined by flow cytometry.

To detect migration of antigen-loaded APCs into lymph nodes, TC-1-bearing wildtype or TLR9^−/−^ mice were treated with cisplatin intraperitoneally, FITC-labeled E7 antigen intratumorally, and/or DNase I intravenously as described in the tumor treatment experiment section. 2 days after the last treatment administration, draining lymph nodes were harvested and homogenized in RPMI-1640 medium in nylon mesh bags. Erythrocytes were lysed with ammonium chloride and washed twice with RPMI-1640 medium. Cells were stained with APC-labeled anti-CD11c mAb, and the frequency of FITC^+^ CD11c^+^ cells was examined by flow cytometry.

### Statistical analysis

All data presented in this study are expressed as mean ± SD and are representative of 3 independent experiments performed. At least 3 samples per group were included in each of these experiments. Flow cytometry data and results of tumor treatment experiments were evaluated by analysis of variance (ANOVA) and the Tukey-Kramer test. Individual data points were compared by Student’s t-test. Event-time distributions for mice were compared by the Kaplan-Meier method and the log-rank test. *P* values < 0.05 were considered significant.

## Data Availability

Data sharing is not applicable to this article as no datasets were generated or analyzed during the current study.

## Abbreviations

APC: Antigen-presenting cell
CTL: CD8+ cytotoxic T lymphocytes
DC: Dendritic cell
E7: Human papillomavirus type 16 Early protein 7
TLR: Toll-like receptor
TME: Tumor microenvironment
